# Consensus statement on obesity management in liver transplantation candidates

**DOI:** 10.1007/s13304-025-02473-x

**Published:** 2026-02-27

**Authors:** David Pacheco Sánchez, Carmen Vinaixa Aunés, Amador Garcia Ruiz de Gordejuela, Cándido Alcázar López, Rosa Álvarez Seoane, Javier Briceño Delgado, David Calatayud Mizrahi, Benjamín Díaz-Zorita Aguilar, Roberto Fernández Santiago, Yiliam Fundora Suárez, Álvaro García Sesma, Carmen García Bernardo, Miguel Angel Gómez Bravo, Concepción Gómez Gavara, Xavier González Argentés, Diego López Guerra, Adolfo López Buenadicha, José Luis Lucena de la Poza, Mónica Mogollón González, Esther Molina Pérez, Custodia Montiel Casado, Javier Padilla Quintana, Pilar Palacios Gasós, Pilar Pinto Fuentes, Pablo Ramírez Romero, Fernando Rotellar Sastre, Lluis Secanella Medayo, Alberto Ventoso Castiñeira, Laura Lladó

**Affiliations:** 1 Liver Transplantation Unit, Department of Surgery, Hospital Universitario Río Hortega, Valladolid, Spain; 2 Hepatology and Liver Transplantation Unit, IIS La Fe and Hospital Universitario y Politécnico La Fe, Valencia, Spain; 3 CIBERehd, Instituto de Salud Carlos III, Madrid, Spain; 4 Esophagogastric and Bariatric Surgery Unit, Hospital Universitario Donostia, The Basque Country University, Donostia-San Sebastian, Spain; 5 Hospital Universitario Reina Sofía, Córdoba, Spain; 6 Hospital Universitario A Coruña, A Coruña, Spain; 7 Hospital Universitario y Politécnico La Fe, Valencia, Spain; 8 Hospital General Universitario Gregorio Marañón, Madrid, Spain; 9 Hospital Universitario Marqués de Valdecilla, Santander, Spain; 10 Hospital Clínic, Barcelona, Spain; 11 Hospital Universitario 12 de Octubre, Madrid, Spain; 12 Hospital Universitario Central de Asturias, Oviedo, Spain; 13 Hospital Universitario Virgen del Rocío, Seville, Spain; 14 Hospital Universitari Vall d’Hebron, Barcelona, Spain; 15 Hospital Universitari Son Espases, Palma, Spain; 16 Hospital Universitario de Badajoz, Badajoz, Spain; 17 Hospital Universitario Ramón y Cajal, Madrid, Spain; 18 Hospital Universitario Puerta de Hierro Majadahonda, Madrid, Spain; 19 Hospital Universitario Virgen de Las Nieves, Granada, Spain; 20 Hospital Clínico Universitario de Santiago, Santiago de Compostela, Spain; 21 Hospital Regional Universitario de Málaga, Málaga, Spain; 22 Hospital Universitario Ntra Sra de La Candelaria, Santa Cruz de Tenerife, Spain; 23 Hospital Clínico Universitario Lozano Blesa, Saragossa, Spain; 24 Hospital Universitario Río Hortega, Valladolid, Spain; 25 Hospital Universitario Virgen de La Arrixaca, Murcia, Spain; 26 Clínica Universidad de Navarra, Pamplona, Spain; 27 Universitario de Cruces, Barakaldo, Spain; 28 Hepatobiliary and Liver Transplantation Unit, Hospital Universitari de Bellvitge, University of Barcelona, Barcelona, Spain

**Keywords:** Liver transplantation and obesity, Bariatric surgery, Cirrhosis, MASLD

## Abstract

The management of obesity in patients with cirrhosis and/or metabolic associated steatotic liver disease (MASLD) is complex. The Spanish Society for Liver Transplantation (SETH) and the Spanish Society for Bariatric Surgery (SECO) have produced a consensus document providing expert recommendations on different topics regarding bariatric surgery and the management of obesity in candidates before and after liver transplantation. A multidisciplinary group of experts composed of a transplant surgeon, a bariatric surgeon and an hepatologist performed a comprehensive review of the literature and identified clinically relevant questions. A preliminary list of recommendations was drafted and further validated, using a modified Delphi approach, by a panel of 25 transplant team delegates. The present consensus statement contains key recommendations alongside the core supporting scientific evidence.

**Graphical Abstract:**

**Figure:**
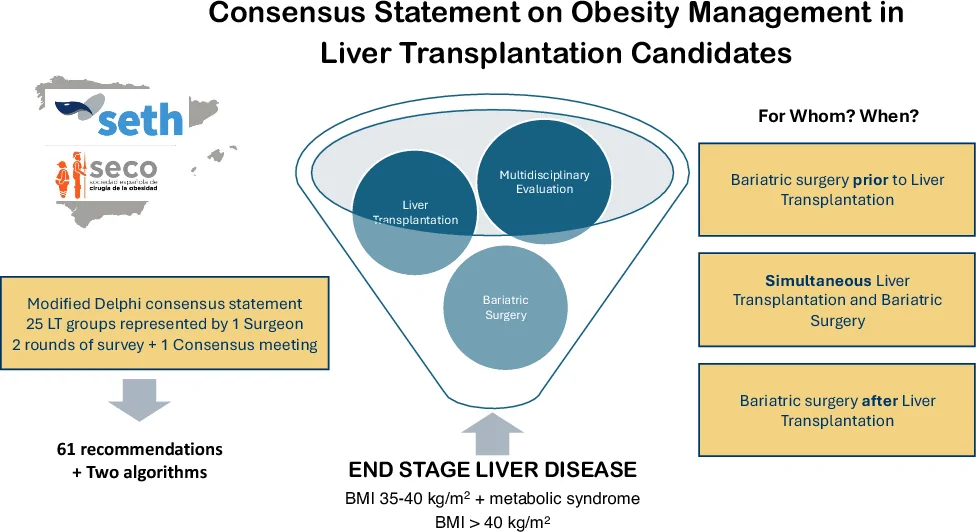

**Supplementary Information:**

The online version contains supplementary material available at https://doi.org/10.1007/s13304-025-02473-x.

## Introduction

According to the World Health Organization (WHO), the prevalence of obesity has tripled globally since 1975, particularly in Western countries, reaching epidemic proportions and being classified as a non-communicable disease [1]. Obesity drives metabolic syndrome (MetS), characterized by hypertension, hypertriglyceridemia, low HDL cholesterol, impaired fasting glucose, and increased abdominal circumference, contributing to conditions like ischemic heart disease, stroke, sleep apnea, osteoarticular disorders, gallstones, and cancers (e.g., colorectal, breast). From a hepatological perspective, obesity is a primary driver of metabolic-associated steatotic liver disease (MASLD). MASLD, frequently advances to metabolic-associated steatohepatitis (MASH), progressive fibrosis, and ultimately end-stage liver disease (ESLD) [2, 3]. Data from the European Liver Transplant Registry (ELTR) and United Network for Organ Sharing (UNOS) [4] indicate that MASLD-related cirrhosis is the fastest-growing indication for liver transplantation (LT). These patients have increased post-transplant complications, particularly cardiovascular events. Among individuals with non-alcoholic MASLD-related cirrhosis, there is an 80% recurrence rate of MASLD within five years following liver transplantation, with elevated post-transplant BMI identified as a significant predictive factor [5].Obesity impacts patients on the LT waiting list, increasing the risk of mortality, particularly in those with class III obesity [6–10]. Patients with obesity are less likely to be accepted for LT [10, 11]. The EASL clinical guideline [12] recommends multidisciplinary discussion for patients with a BMI > 35 kg/m^2^. The American Association for the Study of Liver Diseases (AASLD) [13] classifies class III obesity (BMI > 40 kg/m^2^) as a relative contraindication for LT. An international survey on the management of obese LT candidates revealed that 40% of surveyed groups worldwide set a BMI threshold for contraindicating transplantation: 50% at BMI > 35, 27.3% at BMI > 40, and 22.7% at BMI > 45, while 60% had no BMI limit [6]. In a survey of Spanish LT groups before this consensus and reflecting current practice (unpublished data), 53.8% contraindicated transplantation at BMI > 40, 11.5% at BMI > 35, and 34.6% did not consider BMI for LT eligibility.

Metabolic-bariatric surgery (MBS) is the most effective long-term treatment for severe obesity. MBS in obese MASLD patients induces rapid, significant weight loss and modulates critical metabolic and inflammatory pathways implicated in MASLD pathogenesis [14, 15]. A recent meta-analysis supports these findings, showing significant improvements in histological and biochemical MASLD parameters, endorsing MBS as an effective treatment for MASLD in obese patients [16].

Pre-LT MBS can enhance both LT accessibility and short- and long-term outcomes [17]. However, MBS in ESLD carries risks of bleeding, anastomotic leaks, and hepatic decompensation, with mortality rates of 16–22% in decompensated cirrhosis [18–21]. Evidence is primarily observational due to ethical and logistical barriers to randomized controlled trials (RCTs) [22, 23]. The optimal timing of MBS—pre-LT, simultaneous with LT, or post-LT—remains debated, balancing unique risks and benefits [24, 25].

The Spanish Society for Liver Transplantation (SETH) and the Spanish Society for Bariatric Surgery (SECO) convened a multidisciplinary panel to develop evidence-based recommendations for managing obesity in LT candidates, leveraging MBS to optimize patient selection, procedural safety, and long-term outcomes. Given the limited evidence regarding bariatric surgery in candidates for liver transplantation, a Delphi methodology was chosen.

## Materials and methods

### Panel composition

A coordinating committee, comprising a transplant surgeon, an hepatologist from SETH, and a representative from SECO, assembled 25 delegates including all adult LT units in Spain. Several participants had dual expertise in LT and MBS, enriching the panel’s perspective. Scientific committees from both societies endorsed the methodology and final document.

### Literature review

A comprehensive review searched PubMed, Embase, and Cochrane databases from January 2000 to December 2024, using terms like “bariatric surgery,” “liver transplantation,” “obesity,” “MASLD,” and “cirrhosis.” Over 200 studies were screened, selecting 89 based on methodological rigor and clinical relevance. Inclusion prioritized RCTs, meta-analyses, systematic reviews, and large observational studies. References were appraised using the GRADE system (A = High, robust RCTs; B = Moderate, limited trials or strong observational studies; C = Low, weaker studies or expert opinion) and recommendation strength (1 = Strong, benefits outweigh risks; 2 = Weak, benefits less certain). All the papers reviewed are listed as Supplementary material.

### Delphi process

A modified Delphi process was employed:

- Initial round: anonymous likert-scale questionnaire completed within 15 days, rating statements from “strongly disagree” to “strongly agree.” Consensus required ≥ 70% “agree” or “strongly agree,” with “strong” consensus defined as ≥ 50% “strongly agree” within that 70% or ≥ 90% positive responses with ≥ 35% “strongly agree.”
- Second round: unresolved items were recirculated with aggregated results and anonymized feedback.
- Face-to-face meeting: remaining disagreements were resolved in a live session, achieving full consensus.

**Fig. 1:**
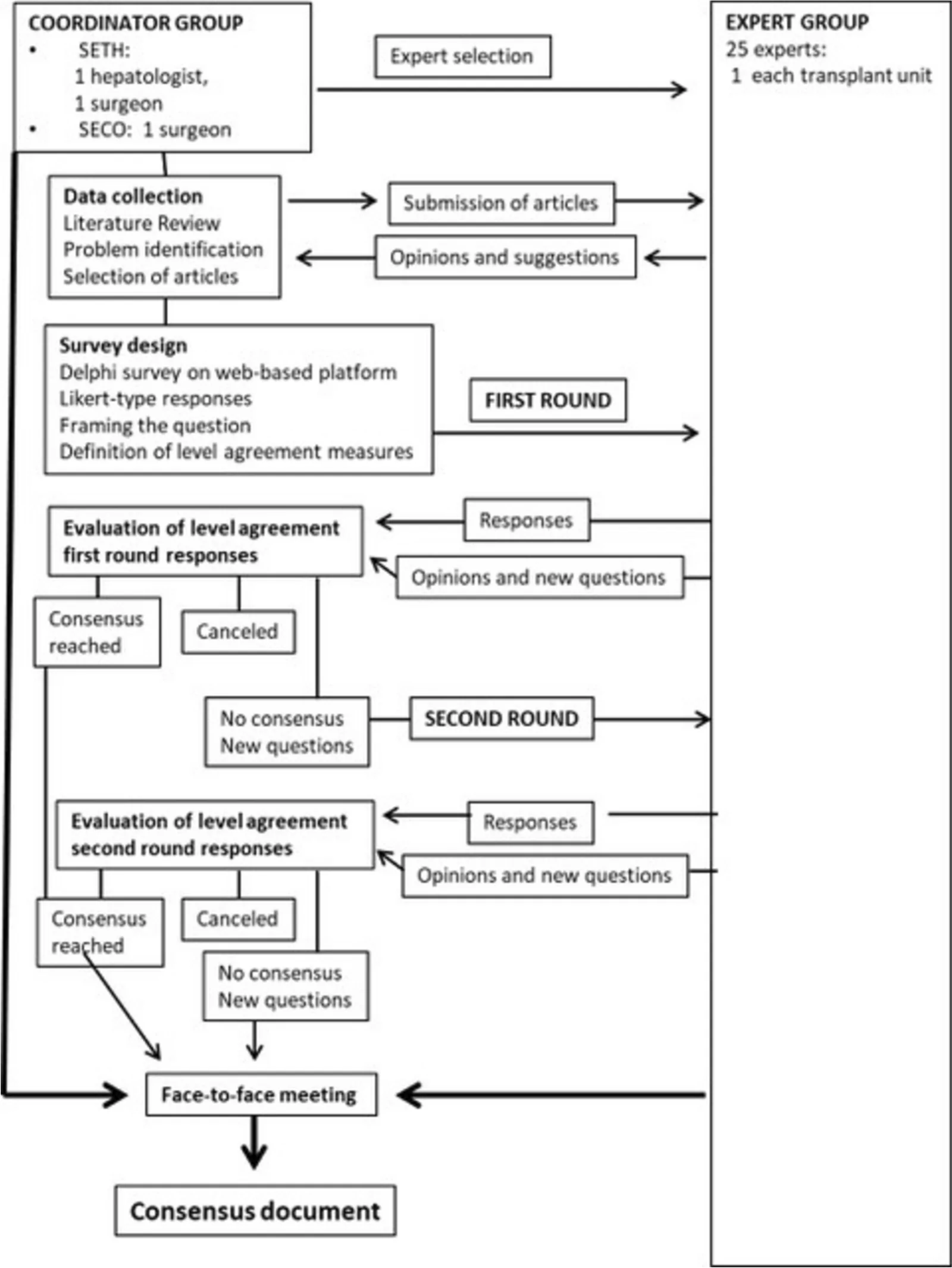
Flow-chart of the consensus process

The process is illustrated in Fig. 1.

### Consensus recommendations

The panel formulated 61 recommendations across four domains: preoperative evaluation, MBS types and timing, post-LT management, and treatment algorithms (Table 1). These recommendations are addressed in more depth in the discussion section. Key recommendations, with supporting evidence, are presented below:

**Table 1:** Summary of recommendations for each item and the corresponding level of evidence

Item	Recommendation	Evidence level	Grade
Evaluation of obese patients prior to LT	Use of “dry BMI” for patients with cirrhosis and ascites	Moderate	Strong
	Indication for patients with obesity and MAFLD to resolve or improve the disease and prevent HCC	Moderate	Strong
	Measures should be implemented to reduce weight and/or evaluate and improve the comorbidities associated with obesity for a better selection of liver transplant (LT) candidates	Moderate	Strong
	The indication for LT in obese cirrhotic patients should be individualized based on a specific BMI threshold	Low	Strong
	In LT candidates with obesity (BMI > 30), an intensive weight loss program and control of associated diseases should be proposed	Moderate	Strong
	Liver transplant centers should coordinate with other specialists (endocrinologists and physiotherapists) to develop specific programs for the evaluation and treatment of obesity	Moderate	Strong
Methods for evaluating cirrhosis and portal hypertension	Pre-operative evaluation to determine the existence of advanced chronic liver disease (cACLD) and clinically significant portal hypertension (CSPH) should be mandatory in patients with MASLD prior to bariatric surgery (BS)	High	Strong
	Performing an upper endoscopy in patients with CSPH should be mandatory to exclude the presence of gastro-esophageal varices prior to performing BS	Moderate	Strong
	The presence of gastro-esophageal varices, collateral circulation, splenomegaly and/or thrombocytopenia are indirect signs of cACLD and CSPH with high positive predictive values	High	Strong
	When no indirect signs of cACLD or CSPH exist, a combination of non-invasive tests (serological and elastography-based) can help determine the fibrosis stage in a high proportion of patients	Moderate	Strong
	When no indirect signs of cACLD or CSPH exist, we propose the use of the FIB-4 index as the first step to exclude cACLD, followed by transient elastography (Fibro Scan^®^) to determine the existence of CSPH	High	Strong
	To diagnose CSPH, spleen elastography may also be used, if available	Moderate	Strong
	If non-invasive tests are not valid or not applicable, invasive procedures such as liver biopsies and/or the measurement of the hepatic venous gradient may be used to determine the existence of cACLD and CSPH, respectively	Moderate	Strong
Type and timing of bariatric surgery	It is recommended that anatomical modifications of BS should be considered when choosing the bariatric technique in these patients	Moderate	Strong
	Obese LT candidates should be advised to follow a supervised weight loss program before being considered for LT in order to lose weight and control or improve comorbidities, especially MAFLD, T2DM, heart disease and hypertension	Moderate	Strong
	Sleeve gastrectomy (SG) should be the bariatric technique of choice in compensated cirrhotic patients with an indication for BS due to its best safety profile, good results in producing weight loss and controlling comorbidity, and ability to enable endoscopic access to the bile duct, if necessary, without altering drug absorption	Moderate	Strong
	BS should not be performed in decompensated cirrhotic patients. Due to their condition and comorbidities, they should be included in weight loss programs based on supervised lifestyle changes alongside other treatments	Moderate	Strong
	Pre-transplant BS should be proposed, mainly in cases with stable liver disease such as Child class A cases with no other contraindications for LT	Moderate	Strong
	Following a risk assessment in a pre-operative study, simultaneous BS and LT can be considered in obese patients with a BMI > 40 in the presence or absence of metabolic comorbidities, regardless of whether they present decompensated cirrhosis or portal hypertension	Low	Weak
	Simultaneous BS and LT should be considered exclusively in the context of centers that have multidisciplinary and coordinated programs between the transplant and obesity management teams	Low	Strong
	Patients undergoing simultaneous BS and LT should undergo exhaustive nutritional supervision to avoid the development or worsening of sarcopenia associated with cirrhosis, protein-calorie malnutrition and micronutrient deficiency	Moderate	Strong
BS and management after LT	BS should be indicated in patients with a BMI > 35 and with significant weight gain associated with post-transplant metabolic syndrome and/or comorbidities such as T2DM and cardiovascular disease	Moderate	Strong
	The safety and efficacy of SG and Roux-en-Y gastric bypass in liver transplant recipients have been demonstrated; therefore, these techniques can be considered the surgeries of choice	High	Strong
	Post-transplant BS should be proposed after considering the timing of the LT and ensuring the absence of significant complications, such as chronic graft dysfunction or rejection, as well as implementing a stable immunosuppressive treatment	High	Strong
	Post-transplant BS should be accompanied by an intensive and multidisciplinary follow-up by a specialized team	High	Strong
	Close monitoring of plasma concentrations of CNIs should be mandatory, as more variability may exist, particularly in patients who have undergone a Roux-en-Y gastric bypass	Low	Strong

### Preoperative evaluation of obesity in LT candidates

- **Dry BMI assessment (B, 1)**: Obesity in cirrhosis should be assessed using “dry BMI” (post-ascites drainage) to ensure accurate body mass representation.
- **Early referral to specialists (B, 1)**: Patients with obesity and ESLD should be referred to weight management and comorbidities assessment at initial hepatology or transplant consultation.
- **MBS for MASLD progression (B, 1)**: MBS should be offered to patients with MASLD to halt disease progression, improve liver function, and potentially avert LT.
- **BMI thresholds (C, 2)**: Patients who are candidates for LT and BMI > 35 kg/m^2^ requires individualized multidisciplinary evaluation. LT candidacy for patients with a BMI > 50 kg/m^2^ obesity is considered a contraindication but subject to case-by-case evaluation. These patients must be evaluated for comorbidities, particularly cardiovascular ones before being listed for liver transplantation.
- **cACLD and CSPH assessment (A, 1)**: Comprehensive assessment for advanced chronic liver disease (cACLD) and clinically significant portal hypertension (CSPH) is mandatory, using non-invasive tests (NITs) (e.g., FIB-4, HEPAMET, transient elastography, spleen stiffness measurement [SSM]) or invasive hepatic venous pressure gradient (HVPG ≥ 10 mmHg) (Table 2) [26–30].
- **Upper endoscopy for varices (A, 1)**: In suspected CSPH, upper endoscopy is mandatory to exclude gastroesophageal varices.
- **Sarcopenic obesity evaluation (B, 1)**: Sarcopenic obesity, combining visceral obesity and muscle loss, should be assessed pre-LT using imaging (e.g., CT) and functional tests.

### Bariatric surgery: techniques and timing

- **Sleeve gastrectomy preference (B, 1)**: Laparoscopic sleeve gastrectomy (SG) is recommended as the primary MBS procedure for LT candidates due to its safety, effective weight loss, comorbidity resolution, and preservation of endoscopic biliary access.
- **RYGB in exceptional cases (B, 2)**: Roux-en-Y gastric bypass (RYGB) is reserved for severe gastroesophageal reflux (e.g., Barrett’s esophagus) in compensated cirrhosis, avoiding use in primary liver cancer due to malabsorption and endoscopic access risks.
- **Pre-LT MBS (B, 1)**: MBS before LT is advised for compensated cirrhosis (Child–Pugh A, MELD < 10, no CSPH) to improve LT eligibility and mitigate metabolic risks.
- **MBS contraindication in decompensated cirrhosis (A, 1)**: MBS is contraindicated in decompensated cirrhosis (CSPH, Child–Pugh B or C, MELD ≥ 15).
- **Simultaneous MBS-LT (C, 2)**: In patients with obesity and decompensated cirrhosis or severe portal hypertension who could not undergo MBS pre-LT, simultaneous MBS (SG) and LT may be considered, in tertiary centers with dual expertise in BS and LT.
- **Post-LT MBS (B, 1)**: Post-LT obesity treatment should include the same steps as in other patients: diet and exercise, pharmacological therapy, and MBS. It is advisable to wait at least 1 year post-LT to reduce the risk of rejection due to immunosuppression changes and to minimize infection risks.
- **Avoid hypoabsorptive procedures (B, 1)**: Hypoabsorptive procedures (e.g., biliopancreatic diversion [BPD], jejunoileal bypass, long-limb RYGB) are discouraged in patients who are candidates for liver transplantation LT due to risks of malabsorption, immunosuppression instability, and acute liver failure.
- **Endoscopic bariatric techniques (C, 2)**: Intragastric balloons may be considered for severe obesity with decompensated cirrhosis contraindicated for LT or MBS, but gastric varices or severe portal hypertensive gastropathy are contraindications.

### Post-LT management following MBS

- **CNI monitoring (B, 1)**: Close monitoring of calcineurin inhibitor (CNI) plasma concentrations is recommended post-MBS, with minimal dose adjustments typically required for SG.
- **Cautious use of derivative MBS (B, 2)**: Derivative techniques (e.g., RYGB) should be used cautiously if biliary access is needed
- **Multidisciplinary follow-up (A, 1)**: Intensive multidisciplinary follow-up is advised post-LT MBS to optimize nutrition, immunosuppression, and comorbidities [32, 53]. This approach mitigates risks of nutritional deficiencies, graft dysfunction, and MASLD recurrence.
- **Pharmacotherapy consideration (C, 2)**: GLP-1/glucagon/GIP receptor simple, dual or triple agonists (e.g., semaglutide, liraglutide, tirzepatide, retatutride) may be considered for weight loss in compensated cirrhosis, pending further ESLD trials [31–33]. These agents show promise in MASLD resolution (e.g., up to 59% NASH resolution with semaglutide), but safety in ESLD remains non-established [34].

### Management algorithms

**Fig. 2:**
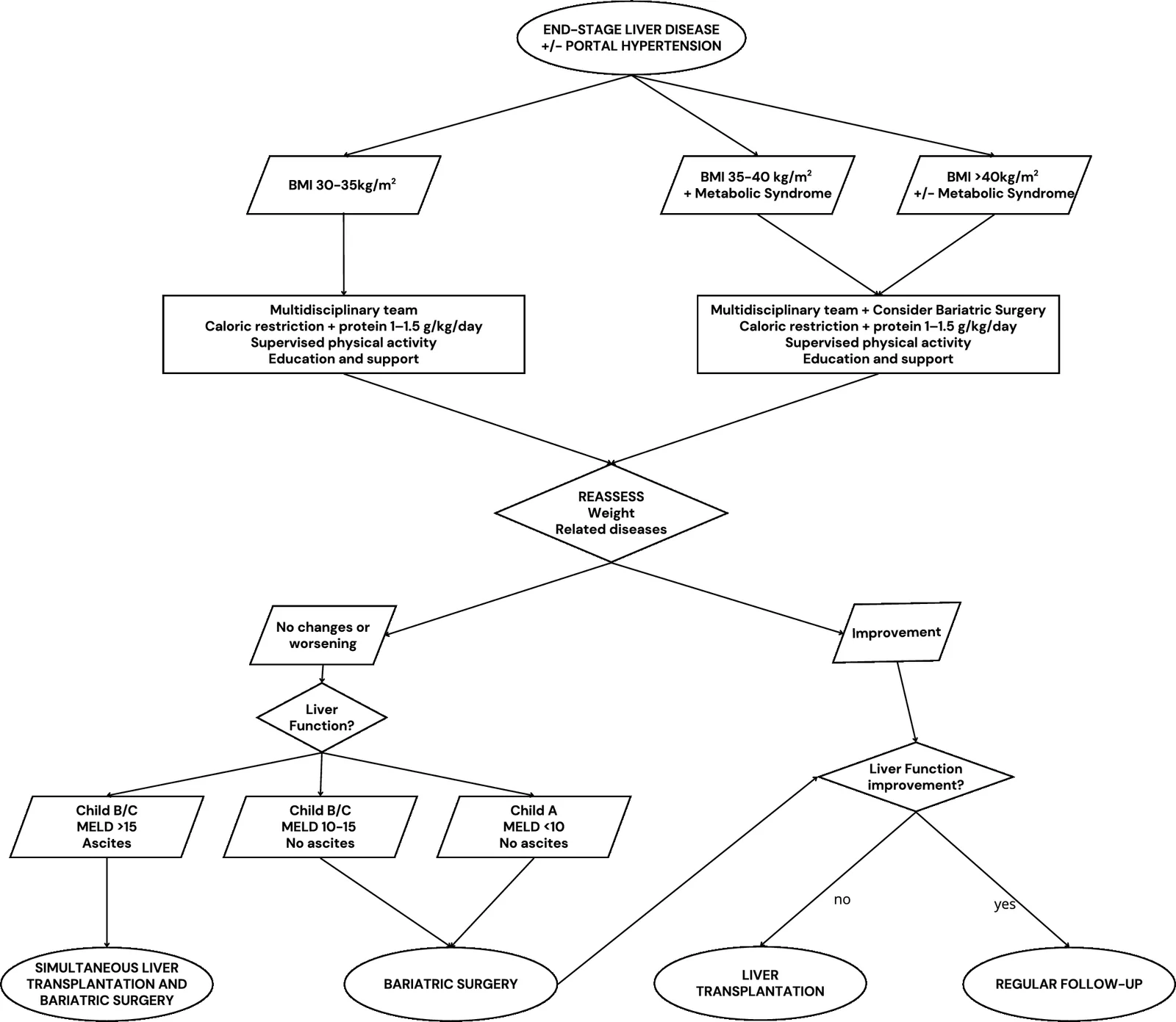
Algorithm for managing obesity in LT candidates

**Fig. 3:**
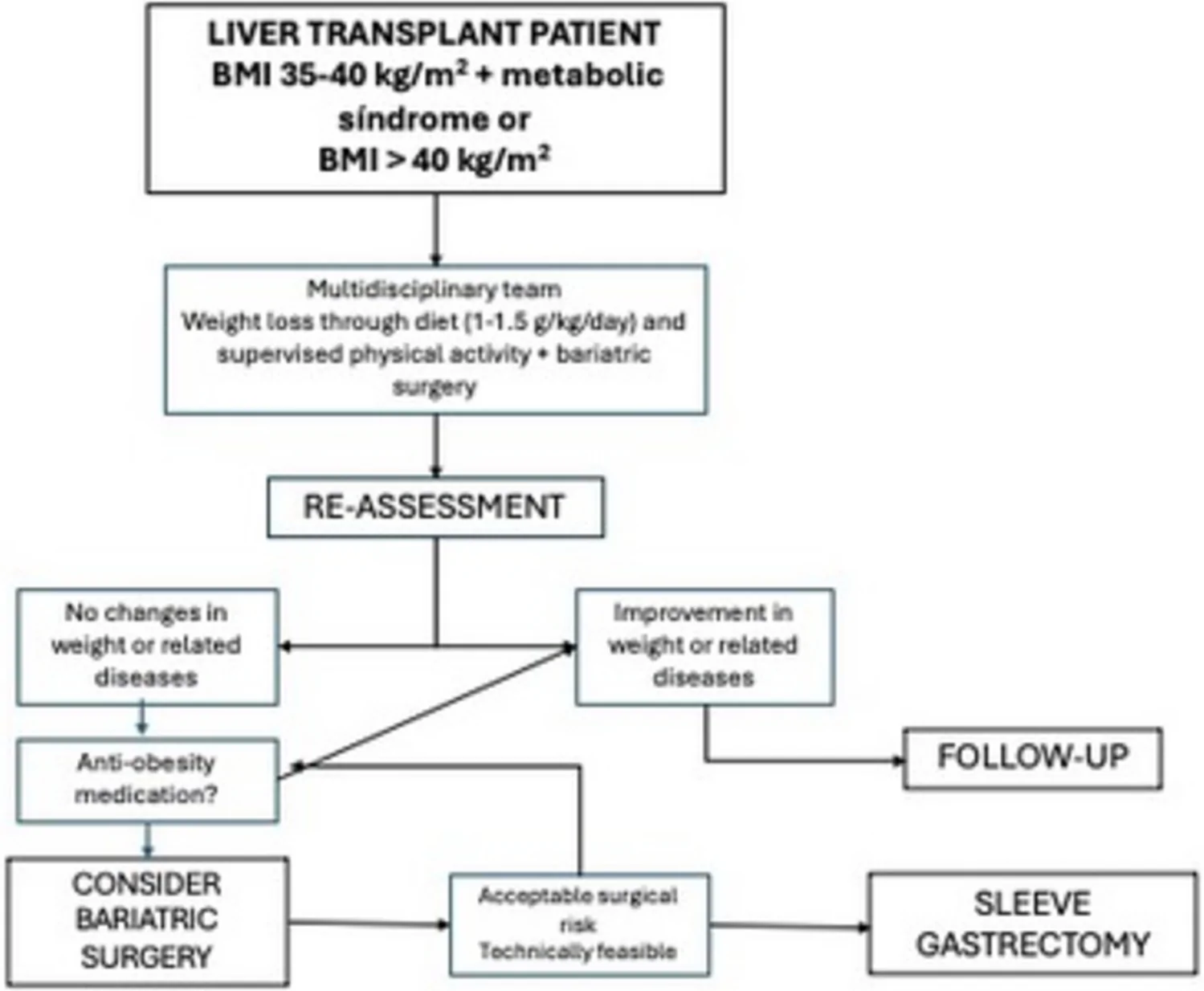
Algorithm for managing obesity in LT recipients

Two algorithms were developed: one for LT candidates (Fig. 2) and one for LT recipients (Fig. 3). BMI cutoffs (> 35 or > 40 kg/m^2^) are indicative, requiring multidisciplinary evaluation to account for comorbidities, sarcopenia, and liver function.

**Table 2:** Summary of the non-invasive tests for evaluating cirrhosis and portal hypertension

Non-invasive tests (NITS)		cACLD diagnosis	CSPH diagnosis	Particularities in MASLD
Imaging ± endoscopy	Presence of esophageal and/or gastric varices/collateral circulation	Yes	Yes	No
	Splenomegaly	Yes	Yes	No
Serological nits	Liver function tests (bilirubin, albumin, INR); Child–Pugh score	Yes	No	Not influenced by steatosis or obesity
	Platelet count	Yes	Yes	No
	Ratio of GOT/GPT > 1	Yes	No	Can increase if associated with alcohol consumption (MetALD)
	FIB-4 score (age, AST, ALT, platelets)	Yes	No	- Good performance and high NPV - Not influenced by obesity - Underestimation if levels/activity of transaminases are normal
	NAFLD score (age, BMI, glycemia, AST, ALT, albumin, platelets)	Yes	No	Influenced by BMI
	HEPAMET score (age, HOMA, AST, albumin, platelets)	Yes	No	- Developed in the Spanish population - Good performance
Elastogaphy-based nits	Liver FibroScan® (transient elastography)	Yes	Yes	- Might be necessary to use the XL probe if the distance between the abdominal wall and the liver > 3.5 cm - Steatosis can lead to an overestimation of the Kpa values
	Spleen elastography	No	Yes	
	Acoustic radiation force impulse (ARFI) elastography	Yes	Yes	Less validated

*cACLD* advanced chronic liver disease, *CSPH* clinically significative portal hypertension, *MASLD* metabolic associated steatotic liver disease

**Table 3:** Influence of BS on immunosuppression levels

	N	Transplant type	Bariatric surgery modality	Findings
Chesu et al. [19]	1	Liver Tx	Jejunoileal bypass	Reduced cyclosporine concentrations (C2)
Rogers et al. [20]	6	4 ESRD & 2 Renal Tx	Laparoscopic sleeve gastrectomy	Pharmacological exposure of tacrolimus and ER‐tac was 50% greater and the MMF and MPS increased 75%‐150%
Al-Nowaylati et al. [21]	7	Liver Tx	RYGB	6/7 patients required an increase in their tacrolimus dosage
Diwan et al. [22]	23	ESRD (no transplant)	Laparoscopic sleeve gastrectomy	No significant changes in immunosuppressant dosing
Yemini et al. [23]	34	Renal, liver, cardiac Tx	RYGB/laparoscopic sleeve gastrectomy	Slight reduction in tacrolimus levels (within therapeutic range). No patient required significant modification in their tacrolimus dosing
Chan et al. [24]	12	Renal Tx	Laparoscopic sleeve gastrectomy	Increase in tacrolimus, ER-tac and MMF (AUC_24_, 46%, 55% and 77%, respectively)
Riordan et al. [25]	4	Cardiac Tx	RYGB/laparoscopic sleeve gastrectomy	Increased variability in CNI plasma levels

*CNI* calcineurin inhibitors, *ESRD* end-stage renal disease, *ER-Tac* extended-release tacrolimus, *MMF* mycophenolate mofetil, *MPA* mycophenolic acid, *Tx* transplant, *RYGB* Roux-en-Y gastric bypass

## Discussion

This consensus statement summarizes the recommendations for the management of obesity in LT candidates under the auspicious and expertise of the Spanish societies for Liver Transplantation and Bariatric Surgery. This is a complex topic with low evidence available and lots of questions without clearness even with contradictories answers. The Delphi methodology is indicated in medicine when seeking expert consensus on complex, uncertain, or evidence-limited topics, such as the development of clinical guidelines, prioritization of interventions, or definition of diagnostic criteria. Its iterative and anonymous nature reduces biases and facilitates the integration of diverse expert opinions through structured questionnaire rounds[35]. After a systematic evaluation of the available literature and 2 rounds of questionnaires, the final consensus meeting produced this document.

### Preoperative evaluation of obesity in LT candidates

Obesity classification, primarily based on Body Mass Index (BMI), is widely utilized in medical literature despite its multiple limitations, and guides eligibility for metabolic-bariatric surgery (MBS). The 2022 American Society for Metabolic and Bariatric Surgery (ASMBS) and International Federation for the Surgery of Obesity and Metabolic Disorders (IFSO) guidelines strongly recommend MBS for patients with BMI ≥ 35 kg/m^2^, irrespective of comorbidities. For class I obesity (BMI 30–34.9 kg/m^2^), MBS is considered in the presence of obesity-related comorbidities, such as T2DM, hypertension, MASLD, dyslipidemia, cardiovascular disease, or obstructive sleep apnea [36].

BMI is a standard metric in the general population, but its applicability in patients with cirrhosis, particularly those with decompensated liver disease and ascites, is limited. No consensus exists on defining obesity in this population. Ascites distorts BMI, leading to misclassification, particularly in decompensated cirrhosis. The European Association for the Study of the Liver (EASL) recommends dry BMI for surgical planning, as it reflects true body composition, critical for LT and MBS eligibility [37].

Lifestyle modifications, including tailored diet and supervised exercise, are foundational for weight loss. Diets should be individualized, with close monitoring to prevent worsening sarcopenia in decompensated patients. The International Society for Hepatic Encephalopathy and Nitrogen Metabolism Consensus and 2020 European Society for Clinical Nutrition and Metabolism guidelines recommend hypocaloric, high-protein diets, avoiding caloric intake below 1000 kcal/day [38, 39]. Compensated cirrhosis patients, with or without portal hypertension, can safely engage in supervised exercise, which may improve portal hypertension, fatigue, nutritional status, muscle mass, and ammonium metabolism. Exercise benefits in decompensated patients are controversial, and unsupervised exercise is generally discouraged [40]. Although lifestyle interventions can achieve > 10% pre-LT weight loss, post-LT weight regain is common.

Pharmacological obesity treatment data in cirrhosis is sparse. Orlistat and liraglutide appear safe in compensated cirrhosis, while emerging glucagon-like peptide-1 receptor analogs (GLP1-R-a) (e.g., semaglutide, tirzepatide) show promise for weight loss and MASLD management [31, 32]. These new drugs have presented significant weight loss in patients without ESLD, probably they will represent a new weapon in the future.

BMI thresholds represent an active debate. The EASL clinical practice guideline[41] advocates multidisciplinary evaluation for patients with BMI > 35 kg/m^2^, while the American Association for the Study of Liver Diseases (AASLD) [13] considers class III obesity (BMI > 40 kg/m^2^) a relative contraindication. An international survey revealed that 40% of global LT centers impose BMI thresholds for contraindicating LT: 50% at BMI > 35, 27.3% at BMI > 40, and 22.7% at BMI > 45, while 60% apply no BMI limit [6].

A recent study of post-LT outcomes in class III obesity recipients found no increased morbidity, mortality, or graft loss compared to normal-weight or class II obesity recipients [42]. However, this cohort comprised only 21 carefully selected patients with minimal comorbidities, optimal donors, and surgeries performed by experienced transplant surgeons. Conversely, a single-center retrospective study reported significantly reduced 5-year patient and graft survival in recipients with BMI > 40 kg/m^2^ [43], though this cohort was small (26 patients) with limited donor data [44]. A meta-analysis of 24 studies involving over 130,000 patients confirmed that class III obesity significantly elevates post-LT mortality risk, with all obesity classes linked to higher postoperative complications [45]. As BMI > 30 increases post-transplant complications, LT programs must adopt strategies to promote weight loss in obese candidates. Beyond BMI, comorbidities, particularly T2DM and cardiovascular disease, drive increased postoperative risks, including respiratory, infectious, and cardiovascular complications, prolonging ICU and hospital stays [46].

Among Spanish LT centers, 53.8% contraindicate LT at BMI > 40, 11.5% at BMI > 35, and 34.6% do not use BMI as a criterion (unpublished data). Our consensus document establishes a relative contraindication with BMI over 50 kg/m^2^, even though this threshold is arbitrary and the known limitations of the BMI as a bad representative of the severity of the disease. The consensus document left an open window for tailored evaluation. Even though obesity is a growing epidemy, there is scarce experience, and we cannot give strong recommendation on this.

Obesity increases mortality risk among LT waiting list patients, particularly those with class III obesity [7, 8]. It exacerbates cirrhosis progression, with BMI independently predicting clinical decompensation. Comorbidities may have a greater impact than obesity itself on waiting list dropout [9]. Patients with class II and III obesity experience longer waiting times and lower LT rates (11% and 29% lower, respectively, compared to normal-weight patients) [10, 11]. High-BMI patients also face increased portal vein thrombosis risk, necessitating vigilant monitoring during the waiting period [47]. Pre-LT weight loss is the optimal strategy to mitigate obesity-related morbidity and mortality in LT candidates.

Patients with obesity are less likely to qualify for LT, yet MBS offers superior, sustainable outcomes in weight loss, comorbidity resolution, and MASLD amelioration, enhancing LT eligibility and outcomes [17]. Evidence suggests that patients with compensated cirrhosis and preserved liver function can safely undergo MBS. Data primarily derive from small prospective series and heterogeneous retrospective studies evaluating various MBS techniques.

### Bariatric surgery: techniques and timing

A systematic review and meta-analysis of 15 studies, encompassing 1,233,602 MBS patients (7424 with cirrhosis), found NASH as the leading cirrhosis etiology, followed by hepatitis C. Portal hypertension was present in 19.3% of cirrhotic patients, with SG being the most common procedure. Follow-up ranged from hospitalization to 9 years. Mean patient age was 50 years, with 80% female. Baseline weight and BMI ranged from 116–149 kg and 39–54 kg/m^2^, respectively. Over half had comorbidities, including MASLD/MASH, T2DM, hypertension, dyslipidemia, and obstructive sleep apnea. MBS achieved significant weight and BMI reductions (-35.1 kg and -15.9 kg/m^2^, respectively), comparable to non-cirrhotic patients. Comorbidity remission rates were 57.9% for MASLD, 58.4% for T2DM, 53.1% for hypertension, and 83.2% for sleep apnea. Liver function improved, with reduced AST and ALT levels. Complications occurred in 20.8% of compensated cirrhotic patients, higher than in non-cirrhotic patients (OR: 2.7, p < 0.05). Mortality was 1.3% overall, 0.9% in compensated cirrhosis, and 18.2% in decompensated cirrhosis. Complication rates were 17.1% for SG, 33.9% for RYGB, 20% for adjustable gastric band, and 14.3% for biliopancreatic diversion, with SG showing the lowest severe (Clavien-Dindo grade III) complication rate (3.9%). Surgery-related mortality was 0.8% for SG, 1.6% for RYGB, 6.7% for adjustable gastric band, and 21.4% for biliopancreatic diversion. MBS was deemed effective for weight loss, comorbidity resolution, and liver damage reversal, with acceptable safety in compensated cirrhosis despite higher risks [19].

A retrospective National Database review of 558,017 MBS patients (3,086 with compensated cirrhosis, 103 with decompensated cirrhosis) found comparable mortality in compensated cirrhosis to non-cirrhotic patients but elevated mortality in decompensated cirrhosis (OR: 83, p < 0.05). Predictors of mortality included advanced age, male sex, RYGB vs. SG (OR: 3.90, p < 0.05), and centers performing < 50 MBS procedures annually (OR: 5.25, p < 0.05). Compensated cirrhosis patients may be considered for MBS with careful patient selection, procedure choice, and high-volume surgical centers [21].

Undiagnosed cirrhosis-stage hepatic steatosis during bariatric surgery can precipitate severe complications, including sepsis, variceal hemorrhage, and hepatic decompensation [48]. Comprehensive preoperative evaluation is essential to stage liver disease and assess for clinically significant portal hypertension (CSPH), which increases complication risks. Direct hepatic venous pressure gradient (HVPG) measurement (≥ 10 mmHg) is the gold standard for CSPH diagnosis but is invasive and not universally available [28]. Indirect methods include gastroscopic visualization of esophageal/gastric varices or imaging evidence of cirrhosis (nodular liver margins, caudate/left lobe hypertrophy, heterogeneous parenchyma) with portal hypertension signs (e.g., portal vein enlargement, umbilical vein recanalization, splenomegaly, collateral circulation, ascites), confirming cirrhosis with CSPH. Non-invasive tests (NITs), including serological and elastographic methods, are valuable for ambiguous cases, offering high negative predictive value for ruling out advanced liver disease and CSPH. In MASLD, HVPG and NITs have limitations. HVPG may be less accurate, showing lower values than other etiologies, and CSPH-compatible values have been reported without histological fibrosis [49]. Serological markers like the NAFLD index are influenced by BMI, increasing false positives in obesity and diabetes. The FIB-4 index, unaffected by obesity, is effective for detecting advanced fibrosis, with values < 1.3 excluding it with > 90% negative predictive value [27]. The HEPAMET Fibrosis Score, developed in Spain, is similarly robust [50].

The Baveno VII consensus guides NIT use for portal hypertension diagnosis. Transient elastography (TE, Fibroscan^®^) values > 25 kPa indicate CSPH in most etiologies (HBV, HCV, alcohol, non-obese MASLD). However, TE values may be falsely elevated in steatosis or obesity, limiting Baveno VII criteria applicability in BMI > 25 patients. The ANTICIPATE-NASH Model, integrating BMI, TE liver stiffness, and platelet count, estimates CSPH but requires further validation [29]. Spleen stiffness measurement (SSM) also shows promise, with a Spanish study of 85 patients (60 with MASLD) demonstrating high accuracy for CSPH diagnosis (< 40.9 and > 49.9 kPa, AUC 0.95) [26].

A 2023 international survey of LT units found 35.9% (n = 52) have MASLD programs, with 75% including bariatric surgeons [6]. Twenty-nine percent (n = 42) combine MBS and LT, with 7.1% (n = 3) performing 21–50 combined procedures and 69% (n = 29) 1–5 over 5 years. Candidates for combined procedures had MASLD and obesity (45.5%), comorbidities (33.8%), or BMI ≥ 35, ≥ 40, or ≥ 45 kg/m^2^ (67.6%, 17.7%, 5.9%, respectively). Pre-LT MBS was preferred (54.5%), with 25.9% favoring simultaneous MBS and 15.4% or 50% post-LT within 3 or 12 months, respectively. SG was preferred by 79.4%.

Spanish LT units reported 46.2% implementing supervised pre-LT weight loss programs, 46.2% individualizing approaches, and 7.7% not requiring weight loss. MBS was considered by 57.7% pre-LT, with 82.6% performing SG, 8.7% RYGB, and 61.5% treating < 10 patients (unpublished data).

Pre-LT MBS risks include sarcopenia, a mortality predictor in cirrhosis, and malnutrition, though liver failure is rare today. A review of 32 post-MBS liver failure cases implicated obsolete jejunoileal bypass (50%) and biliopancreatic diversion (44%), with one RYGB case and none for SG [51]. RYGB patients with MASLD may experience transient liver function deterioration compared to SG, resolving by 12 months [52]. RYGB is associated with higher morbidity and mortality than SG, particularly in LT candidates [53, 54]. A meta-analysis showed comparable comorbidity resolution between SG and RYGB, with SG being safer despite less excess weight loss [55].

SG’s preservation of gastroduodenal transit facilitates endoscopic biliary access and treatment of variceal or ulcer-related bleeding, unlike RYGB. SG also reduces malnutrition risk, enhancing immunosuppressive drug absorption (e.g., mycophenolate mofetil) [56].

Iannelli et al. compared 39 LT patients with prior MBS to 1,798 LT patients with obesity and without MBS, finding no differences in mortality, re-LT, rehospitalization, or costs [23]. A systematic review of five studies with comparative arms (162 MBS + LT vs. 1,426 LT-only patients) reported no significant differences in patient/graft survival or postoperative morbidity [49]. Four studies evaluated pre-LT MBS (n = 133), one simultaneous MBS + LT (n = 26), predominantly using RYGB (n = 82) and SG (n = 32). Morbidity included complications within 30 days post-LT (e.g., biliary stenosis, hemorrhage, infection). Simultaneous MBS + LT showed higher NASH recurrence in non-MBS patients. Comparable outcomes in MBS patients, despite worse baseline health and comorbidities, underscore MBS’s value in enabling LT candidacy [57, 58].

Simultaneous MBS during LT promotes weight loss and mitigates immunosuppression-exacerbated comorbidities (e.g., diabetes, dyslipidemia, hypertension). It is suitable for patients with obesity and decompensated cirrhosis or severe portal hypertension ineligible for pre-LT MBS. The Mayo Clinic reported 13 simultaneous LT + SG cases, with no deaths but two complications (staple line leak, excessive weight loss). Non-SG patients developed BMI > 35, diabetes, and steatosis in 60%, 34%, and 20% of cases, respectively, vs. 0% in SG patients, with 35% vs. 4% total body weight loss at 3 years [54]. Small series confirm simultaneous LT + SG’s safety, minimal adverse effects, and benefits in weight loss, comorbidity resolution, and MASLD prevention [25, 59]. Though evidence is limited, simultaneous SG demonstrates acceptable safety and efficacy in experienced centers. Logistical challenges include coordinating bariatric surgical teams during LT.

### Post-LT management following MBS

Post-LT obesity management mirrors general approaches: diet, exercise, pharmacotherapy, and MBS. Post-LT MBS is technically challenging and carries higher morbidity than in non-LT patients. Waiting at least 1 year post-LT minimizes rejection and infection risks due to immunosuppression [60]. Steroid use, common in the first 3–6 months post-LT, increases 30-day MBS morbidity/mortality [61]. SG post-LT shows comparable efficacy and safety to non-transplanted populations in small series [62–64], while RYGB reports higher morbidity/mortality. A meta-analysis found post-LT MBS had a 16% major complication rate, attributed to technical complexity and patient frailty [55]. Post-LT MBS is viable for de novo obesity, though adhesions and bleeding complicate reoperations. Only 15.4% of international survey respondents preferred post-LT MBS [6], with minimal Spanish support (< 5%) (unpublished data).

MBS may alter immunosuppressive drug bioavailability [43, 65]. Tacrolimus and cyclosporine are absorbed primarily in the proximal duodenum, where p-glycoprotein and CYP3A mediate first-pass metabolism. RYGB reduces absorption surface, while adjustable gastric banding accelerates gastric emptying. Limited data from case series across organ transplants show variable but generally non-significant plasma concentration changes, with minimal dose adjustments and no rejection cases. Close monitoring of calcineurin inhibitor levels is mandatory. (Table 3)

Comprehensive multidisciplinary care is crucial following post-LT MBS to enhance nutritional status, immunosuppression management, and comorbidity control [54, 66]. This strategy reduces the risks of malnutrition, graft impairment, and MASLD recurrence.

### Limitations

This consensus relies predominantly on observational studies and small case series, particularly for simultaneous BS-LT, due to ethical and logistical barriers to RCTs. This results in low-quality evidence (GRADE C) for several recommendations, limiting their generalization and robustness. While a comprehensive literature review of over 200 studies was conducted, the absence of RTCs restricts the depth of evidence synthesis and may introduce selection bias, as the panel prioritized studies relevant to LT and BS. The use of the Delphi methodology is specifically designed for cases where the scientific response to a problem is based on limited evidence [35].

This consensus represents the experience and expertise of the Spanish surgeons and hepatologists. Even most of the recommendations are based in the literature, there might be some conclusions that may be biased by local practices and previous experiences, so we acknowledge that some of the recommendations may not be applicable at other countries.

Finally, the consensus does not fully address the long-term outcomes of novel pharmacotherapies, such as GLP-1a, due to limited evidence in ESLD and LT populations. While these agents show promise in managing obesity and MALFD, their safety profile in advanced liver disease remains uncertain, requiring further investigation.

These limitations highlight critical research gaps, including the need for prospective studies, multicenter registries, and clinical trials to strengthen the evidence base and refine recommendations for diverse clinical and geographical contexts.

## Conclusion

This SETH-SECO consensus provides a framework for managing obesity in LT candidates, emphasizing SG, rigorous preoperative evaluation, and multidisciplinary care. Recommendations balance scientific rigor with practical considerations to optimize outcomes for patients with obesity. Research priorities include longitudinal studies on future research is needed to strengthen the evidence base and expand access for LT candidates with obesity.

## Supplementary Information

Below is the link to the electronic supplementary material.

## Data Availability

Not applicable
