# Supplementary material for: Consensus statement on obesity management in liver transplantation candidates
Source: Updates Surg. 2026 Feb 27;78(5):1901–14. doi: 10.1007/s13304-025-02473-x (PMC13538192; doi:10.1007/s13304-025-02473-x)
Supplement: Supplementary file 1 [file 13304_2025_2473_MOESM1_ESM.docx]

**PRELIMINARY SURVEY MAP OF THE SITUATION IN SPAIN**

25 participating groups: 100% of respondents

**Do you think morbid obesity should be an absolute contraindication for liver transplantation?**

No: 50%

Depending on other factors: 46.2%

Yes: 3.8%

**In your unit, what are the key factors to consider when evaluating an obese patient for a liver transplant? (Select all that apply)**

Medically related complications: 88.5%

BMI: 84.6%

**Do you think obesity could negatively influence the results of a liver transplant?**

Yes: 92.3%

**At which BMI would your unit consider liver transplantation to be contraindicated if the patient fails to lose weight?**

BMI > 40: 53.8%

We do not take the BMI into account: 34.6%

BMI > 35-40: 11.5%

**In your unit, should obese patients follow a supervised weight loss program before being considered for a liver transplant?**

Yes: 46.2%

It depends on each case: 46.2%

Weight loss should not be a requirement: 7.6%

**Is the decision to perform a liver transplant in an obese patient in your unit based on a multidisciplinary approach involving several medical specialists (endocrinologists and bariatric surgeons) in addition to those who make up the usual committee?**

Yes: 69.4%

No: 30.6%

**Should bariatric surgery be considered a strategy to reduce weight in morbidly obese patients before they are considered for a liver transplant?**

Yes: 57.7%

No: 42.3%

**When does your unit indicate bariatric surgery in an obese patient who is a candidate for a liver transplant?**

According to the patient's characteristics: 57.7%

We do not indicate bariatric surgery: 19.2%

Prior to liver transplantation: 11.5%

After liver transplantation: 5.8%

We do not indicate bariatric surgery in these patients: 5.8%

**What technique is most frequently used in your unit in these patients?**

Sleeve gastrectomy: 82.6%

Gastric bypass: 8.7%

Other: 8.7%

**How many patients have undergone bariatric surgery in your unit regardless of the time of the transplant?**

Fewer than 10: 61.5%

None: 30.8%

10-50: 7.7%

**Do you consider that a consensus among liver transplant groups is necessary to reduce clinical variability in the approach to obese patients who are candidates for liver transplantation?**

Yes: 100%
