# Supplementary material for: Consensus statement on obesity management in liver transplantation candidates
Source: Updates Surg. 2026 Feb 27;78(5):1901–14. doi: 10.1007/s13304-025-02473-x (PMC13538192; doi:10.1007/s13304-025-02473-x)
Supplement: Supplementary file 2 [file 13304_2025_2473_MOESM2_ESM.docx]

- Addeo P, Cesaretti M, Anty R, Iannelli A (2019) Liver transplantation for bariatric surgery-related liver failure: a systematic review of a rare condition. Surg Obes Relat Dis 15:1394–1401. https://doi.org/10.1016/j.soard.2019.06.002
- Al-Nowaylati A-RR, Al-Haddad BJSS, Dorman RB, et al (2013) Gastric bypass after liver transplantation. Liver Transplantation 19:1324–1329. https://doi.org/10.1002/lt.23734
- Amodio P, Bemeur C, Butterworth R, et al (2013) The nutritional management of hepatic encephalopathy in patients with cirrhosis: International Society for Hepatic Encephalopathy and Nitrogen Metabolism Consensus. Hepatology 58:325–36. https://doi.org/10.1002/hep.26370
- Ampuero J, Pais R, Aller R, et al (2020) Development and Validation of Hepamet Fibrosis Scoring System–A Simple, Noninvasive Test to Identify Patients With Nonalcoholic Fatty Liver Disease With Advanced Fibrosis. Clinical Gastroenterology and Hepatology 18:216-225.e5. https://doi.org/10.1016/j.cgh.2019.05.051
- Andalib A, Aminian A, Khorgami Z, et al (2016) Early Postoperative Outcomes of Primary Bariatric Surgery in Patients on Chronic Steroid or Immunosuppressive Therapy. Obes Surg 26:1479–1486. https://doi.org/10.1007/s11695-015-1923-0
- Angeles PC, Robertsen I, Seeberg LT, et al (2019) The influence of bariatric surgery on oral drug bioavailability in patients with obesity: A systematic review. Obesity Reviews 20:1299–1311. https://doi.org/10.1111/obr.12869
- Antipass A, Austin A, Awad S, et al (2020) Evaluation of Liver Function Tests and Risk Score Assessment to Screen Patients for Significant Liver Disease Prior to Bariatric and Metabolic Surgery. Obes Surg 30:2840–2843. https://doi.org/10.1007/s11695-020-04486-4
- Arab JP, Díaz LA, Dirchwolf M, et al (2021) NAFLD: Challenges and opportunities to address the public health problem in Latin America. Ann Hepatol 24:100359. https://doi.org/10.1016/j.aohep.2021.100359
- Are VS, Knapp SM, Banerjee A, et al (2020) Improving Outcomes of Bariatric Surgery in Patients With Cirrhosis in the United States: A Nationwide Assessment. Am J Gastroenterol 115:1849–1856. https://doi.org/10.14309/ajg.0000000000000911
- Ayala R, Grande S, Bustelos R, et al (2012) Obesity is an independent risk factor for pre-transplant portal vein thrombosis in liver recipients. BMC Gastroenterol 12:114. https://doi.org/10.1186/1471-230X-12-114
- Bai J, Jia Z, Chen Y, et al (2022) Bariatric Surgery is Effective and Safe for Obese Patients with Compensated Cirrhosis: A Systematic Review and Meta‐Analysis. World J Surg 46:1122–1133. https://doi.org/10.1007/s00268-021-06382-z
- Barone M, Viggiani MT, Losurdo G, et al (2017) Systematic review with meta-analysis: post-operative complications and mortality risk in liver transplant candidates with obesity. Aliment Pharmacol Ther 46:236–245
- Berzigotti A, Albillos A, Villanueva C, et al (2017) Effects of an intensive lifestyle intervention program on portal hypertension in patients with cirrhosis and obesity: The SportDiet study. Hepatology 65:1293–1305. https://doi.org/10.1002/hep.28992
- Berzigotti A, Garcia-Tsao G, Bosch J, et al (2011) Obesity is an independent risk factor for clinical decompensation in patients with cirrhosis. Hepatology 54:555–561. https://doi.org/10.1002/hep.24418
- Berzigotti A, Reig M, Abraldes JG, et al (2015) Portal hypertension and the outcome of surgery for hepatocellular carcinoma in compensated cirrhosis: a systematic review and meta-analysis. Hepatology 61:526–36. https://doi.org/10.1002/hep.27431
- Billeter AT, Senft J, Gotthardt D, et al (2016) Combined Non-alcoholic Fatty Liver Disease and Type 2 Diabetes Mellitus: Sleeve Gastrectomy or Gastric Bypass?—a Controlled Matched Pair Study of 34 Patients. Obes Surg 26:1867–1874. https://doi.org/10.1007/s11695-015-2006-y
- Bischoff SC, Bernal W, Dasarathy S, et al (2020) ESPEN practical guideline: Clinical nutrition in liver disease. Clin Nutr 39:3533–3562. https://doi.org/10.1016/j.clnu.2020.09.001
- Bronswijk M, Jaekers J, Vanella G, et al (2022) Umbilical hernia repair in patients with cirrhosis: who, when and how to treat. Hernia 26:1447–1457. https://doi.org/10.1007/s10029-022-02617-7
- Bustamante-Bernal MA, Chavez LO, Zuckerman MJ (2022) Endoscopic Bariatric Interventions in Patients with Chronic Liver Disease. Clin Liver Dis 26:139–148. https://doi.org/10.1016/j.cld.2021.08.005
- Chan G, Hajjar R, Boutin L, et al (2020) Prospective study of the changes in pharmacokinetics of immunosuppressive medications after laparoscopic sleeve gastrectomy. American Journal of Transplantation 20:582–588. https://doi.org/10.1111/ajt.15602
- Chenhsu R-Y, Wu Y, Katz D, Rayhill S (2003) Dose-Adjusted Cyclosporine C2 in a Patient with Jejunoileal Bypass as Compared to Seven Other Liver Transplant Recipients. Ther Drug Monit 25:665–670. https://doi.org/10.1097/00007691-200312000-00004
- Chierici A, Alromayan M, De Fatico S, et al (2023) Is bariatric surgery safer before, during, or after liver transplantation? A systematic review and meta-analysis. Journal of Liver Transplantation 9:100139. https://doi.org/10.1016/j.liver.2023.100139
- Chierici A, Bulsei J, Castaldi A, et al (2022) Clinical and Economic Impact of Bariatric Surgery Post Liver Transplantation: a Nationwide, Population-Based Retrospective Study. Obes Surg 32:2548–2555. https://doi.org/10.1007/s11695-022-06120-x
- Choudhary NS, Saigal S, Saraf N, et al (2013) Innovative approach using an intragastric balloon for weight loss in a morbidly obese patient undergoing liver transplantation. Liver Transplantation 19:235. https://doi.org/10.1002/lt.23567
- Conzen KD, Vachharajani N, Collins KM, et al (2015) Morbid obesity in liver transplant recipients adversely affects longterm graft and patient survival in a single‐institution analysis. HPB 17:251–257. https://doi.org/10.1111/hpb.12340
- Diwan TS, Rice TC, Heimbach JK, Schauer DP (2018) Liver Transplantation and Bariatric Surgery: Timing and Outcomes. Liver Transplantation 24:1280–1287. https://doi.org/10.1002/lt.25303
- Eisenberg D, Shikora SA, Aarts E, et al (2023) 2022 American Society of Metabolic and Bariatric Surgery (ASMBS) and International Federation for the Surgery of Obesity and Metabolic Disorders (IFSO) Indications for Metabolic and Bariatric Surgery. Obes Surg 33:3–14. https://doi.org/10.1007/s11695-022-06332-1
- Eslam M, Newsome PN, Sarin SK, et al (2020) A new definition for metabolic dysfunction-associated fatty liver disease: An international expert consensus statement. J Hepatol 73:202–209. https://doi.org/10.1016/j.jhep.2020.03.039
- Esquivel CM, Garcia M, Armando L, et al (2018) Laparoscopic Sleeve Gastrectomy Resolves NAFLD: Another Formal Indication for Bariatric Surgery? Obes Surg 28:4022–4033. https://doi.org/10.1007/s11695-018-3466-7
- European Association for the Study of the Liver (2019) EASL Clinical Practice Guidelines on nutrition in chronic liver disease. J Hepatol 70:172–193. https://doi.org/10.1016/j.jhep.2018.06.024
- European Association for the Study of the Liver (2024) EASL Clinical Practice Guidelines on liver transplantation. J Hepatol 81:1040–1086. https://doi.org/10.1016/j.jhep.2024.07.032
- Fussner LA, Heimbach JK, Fan C, et al (2015) Cardiovascular disease after liver transplantation: When, What, and Who Is at Risk. Liver Transplantation 21:889–896. https://doi.org/10.1002/lt.24137
- García-Sesma A, Calvo J, Manrique A, et al (2019) Morbidly Obese Patients Awaiting Liver Transplantation—Sleeve Gastrectomy: Safety and Efficacy From a Liver Transplant Unit Experience. Transplant Proc 51:33–37. https://doi.org/10.1016/j.transproceed.2018.01.060
- Geerts WH, Bergqvist D, Pineo GF, et al (2008) Prevention of venous thromboembolism: American College of Chest Physicians evidence-based clinical practice guidelines (8th edition). Chest 133:381–453. https://doi.org/10.1378/chest.08-0656
- Gunturu NS, Castillo-Larios R, Bowers S, et al (2022) Combined Sleeve Gastrectomy with Liver Transplant in Patients with Obesity: a Feasibility Study. Obes Surg 32:3600–3604. https://doi.org/10.1007/s11695-022-06289-1
- Hakeem AR, Cockbain AJ, Raza SS, et al (2013) Increased morbidity in overweight and obese liver transplant recipients: A single-center experience of 1325 patients from the United Kingdom. In: Liver Transplantation. pp 551–562
- Haldar D, Kern B, Hodson J, et al (2019) Outcomes of liver transplantation for non-alcoholic steatohepatitis: A European Liver Transplant Registry study. J Hepatol 71:313–322. https://doi.org/10.1016/j.jhep.2019.04.011
- Halegoua-De Marzio DL, Wong S-Y, Fenkel JM, et al (2016) Listing Practices for Morbidly Obese Patients at Liver Transplantation Centers in the United States. Exp Clin Transplant 14:646–649. https://doi.org/10.6002/ect.2015.0247
- Heimbach JK, Watt KDS, Poterucha JJ, et al (2013) Combined Liver Transplantation and Gastric Sleeve Resection for Patients With Medically Complicated Obesity and End-Stage Liver Disease. American Journal of Transplantation 13:363–368. https://doi.org/10.1111/j.1600-6143.2012.04318.x
- Idriss R, Hasse J, Wu T, et al (2019) Impact of Prior Bariatric Surgery on Perioperative Liver Transplant Outcomes. Liver Transplantation 25:217–227. https://doi.org/10.1002/lt.25368
- Kalinowski P, Paluszkiewicz R, Ziarkiewicz-Wróblewska B, et al (2017) Liver Function in Patients with Nonalcoholic Fatty Liver Disease Randomized to Roux-en-Y Gastric Bypass Versus Sleeve Gastrectomy. Ann Surg 266:738–745. https://doi.org/10.1097/SLA.0000000000002397
- Kardashian AA, Dodge JL, Roberts J, Brandman D (2018) Weighing the risks: Morbid obesity and diabetes are associated with increased risk of death on the liver transplant waiting list. Liver International 38:553–563. https://doi.org/10.1111/liv.13523
- Khoraki J, Katz MG, Funk LM, et al (2016) Feasibility and outcomes of laparoscopic sleeve gastrectomy after solid organ transplantation. Surgery for Obesity and Related Diseases 12:75–83. https://doi.org/10.1016/j.soard.2015.04.002
- Laursen TL, Hagemann CA, Wei C, et al (2019) Bariatric surgery in patients with non-alcoholic fatty liver disease - from pathophysiology to clinical effects. World J Hepatol 11:138–149. https://doi.org/10.4254/wjh.v11.i2.138
- Lee HA, Kim HY (2023) Therapeutic Mechanisms and Clinical Effects of Glucagon-like Peptide 1 Receptor Agonists in Nonalcoholic Fatty Liver Disease. Int J Mol Sci 24:. https://doi.org/10.3390/ijms24119324
- Loomba R, Hartman ML, Lawitz EJ, et al (2024) Tirzepatide for Metabolic Dysfunction-Associated Steatohepatitis with Liver Fibrosis. N Engl J Med 391:299–310. https://doi.org/10.1056/NEJMoa2401943
- Lopez-Lopez V, Ruiz-Manzanera JJ, Eshmuminov D, et al (2021) Are We Ready for Bariatric Surgery in a Liver Transplant Program? A Meta-Analysis. Obes Surg 31:1214–1222. https://doi.org/10.1007/s11695-020-05118-7
- Lorico S, Colton B (2020) Medication management and pharmacokinetic changes after bariatric surgery. Can Fam Physician 66:409–416
- Mahmud N, Fricker Z, Hubbard RA, et al (2021) Risk Prediction Models for Post‐Operative Mortality in Patients With Cirrhosis. Hepatology 73:204–218. https://doi.org/10.1002/hep.31558
- Martin P, DiMartini A, Feng S, et al (2014) Evaluation for liver transplantation in adults: 2013 practice guideline by the American Association for the Study of Liver Diseases and the American Society of Transplantation. Hepatology 59:1144–1165. https://doi.org/10.1002/hep.26972
- Mathur A, Franco ES, Leone JP, et al (2013) Obesity portends increased morbidity and earlier recurrence following liver transplantation for hepatocellular carcinoma. HPB 15:504–510. https://doi.org/10.1111/j.1477-2574.2012.00602.x
- McPherson S, Stewart SF, Henderson E, et al (2010) Simple non-invasive fibrosis scoring systems can reliably exclude advanced fibrosis in patients with non-alcoholic fatty liver disease. Gut 59:1265–1269. https://doi.org/10.1136/gut.2010.216077
- Miao L, Targher G, Byrne CD, et al (2024) Current status and future trends of the global burden of MASLD. Trends in Endocrinology & Metabolism 35:697–707. https://doi.org/10.1016/j.tem.2024.02.007
- Moctezuma-Velazquez C, Márquez-Guillén E, Torre A (2019) Obesity in the Liver Transplant Setting. Nutrients 11:2552. https://doi.org/10.3390/nu11112552
- Morris MC, Jung AD, Kim Y, et al (2019) Delayed Sleeve Gastrectomy Following Liver Transplantation: A 5-Year Experience. Liver Transpl 25:1673–1681. https://doi.org/10.1002/lt.25637
- Mosko JD, Nguyen GC (2011) Increased perioperative mortality following bariatric surgery among patients with cirrhosis. Clin Gastroenterol Hepatol 9:897–901. https://doi.org/10.1016/j.cgh.2011.07.007
- Mumtaz K, Lipshultz H, Jalil S, et al (2020) Bariatric Surgery in Patients with Cirrhosis: Careful Patient and Surgery-Type Selection Is Key to Improving Outcomes. Obes Surg 30:3444–3452. https://doi.org/10.1007/s11695-020-04583-4
- Murag S, Ahmed A, Kim D (2021) Recent Epidemiology of Nonalcoholic Fatty Liver Disease. Gut Liver 15:206–216. https://doi.org/10.5009/gnl20127
- Muthukrishnan S, Zhou J, Wang R, et al (2025) Challenges and strategies for optimizing liver transplantation outcomes in morbidly obese cirrhotic patients: a narrative review. BMC Surg 25:267. https://doi.org/10.1186/s12893-025-02886-w
- Newsome PN, Buchholtz K, Cusi K, et al (2021) A Placebo-Controlled Trial of Subcutaneous Semaglutide in Nonalcoholic Steatohepatitis. New England Journal of Medicine 384:1113–1124. https://doi.org/10.1056/NEJMoa2028395
- Odriozola A, Puente Á, Cuadrado A, et al (2023) High accuracy of spleen stiffness measurement in diagnosing clinically significant portal hypertension in metabolic‐associated fatty liver disease. Liver International 43:1446–1457. https://doi.org/10.1111/liv.15561
- Peck JR, Latchana N, Michaels A, et al (2017) Diagnosis of morbid obesity may not impact healthcare utilization for orthotopic liver transplantation: A propensity matched study. World J Hepatol 9:595. https://doi.org/10.4254/wjh.v9.i12.595
- Pons M, Augustin S, Scheiner B, et al (2021) Noninvasive Diagnosis of Portal Hypertension in Patients With Compensated Advanced Chronic Liver Disease. Am J Gastroenterol 116:723–732. https://doi.org/10.14309/ajg.0000000000000994
- Qadri S, Ahlholm N, Lønsmann I, et al (2022) Obesity Modifies the Performance of Fibrosis Biomarkers in Nonalcoholic Fatty Liver Disease. J Clin Endocrinol Metab 107:e2008–e2020. https://doi.org/10.1210/clinem/dgab933
- Ri M, Aikou S, Seto Y (2018) Obesity as a surgical risk factor. Ann Gastroenterol Surg 2:13–21. https://doi.org/10.1002/ags3.12049
- Riordan M, Oreschak K, Peters LL, et al (2021) Bariatric Surgery Is Associated With Decreased Calcineurin Inhibitor Time in Therapeutic Range After Heart Transplantation. Transplant Proc 53:681–685. https://doi.org/10.1016/j.transproceed.2021.01.020
- Rodrigues SG, Montani M, Guixé-Muntet S, et al (2019) Patients With Signs of Advanced Liver Disease and Clinically Significant Portal Hypertension Do Not Necessarily Have Cirrhosis. Clin Gastroenterol Hepatol 17:2101-2109.e1. https://doi.org/10.1016/j.cgh.2018.12.038
- Saeed N, Glass L, Sharma P, et al (2019) Incidence and Risks for Nonalcoholic Fatty Liver Disease and Steatohepatitis Post-liver Transplant: Systematic Review and Meta-analysis. Transplantation 103:e345–e354. https://doi.org/10.1097/TP.0000000000002916
- Safavi D, Creavin B, Gallagher TK, Kelly ME (2022) The role of bariatric surgery in liver transplantation: timing and type. Langenbecks Arch Surg 407:3249–3258. https://doi.org/10.1007/s00423-022-02606-5
- Sarno G, Schiavo L, Calabrese P, et al (2022) The Impact of Bariatric-Surgery-Induced Weight Loss on Patients Undergoing Liver Transplant: A Focus on Metabolism, Pathophysiological Changes, and Outcome in Obese Patients Suffering NAFLD-Related Cirrhosis. J Clin Med 11:5293. https://doi.org/10.3390/jcm11185293
- Sasaki A, Nitta H, Otsuka K, et al (2014) Bariatric surgery and non-alcoholic Fatty liver disease: current and potential future treatments. Front Endocrinol (Lausanne) 5:164. https://doi.org/10.3389/fendo.2014.00164
- Schlansky B, Naugler WE, Orloff SL, Enestvedt CK (2016) Higher Mortality and Survival Benefit in Obese Patients Awaiting Liver Transplantation. Transplantation 100:2648–2655. https://doi.org/10.1097/TP.0000000000001461
- Segev DL, Thompson RE, Locke JE, et al (2008) Prolonged waiting times for liver transplantation in obese patients. Ann Surg 248:863–70. https://doi.org/10.1097/SLA.0b013e31818a01ef
- Segna D, Mendoza YP, Lange NF, et al (2023) Non-invasive tools for compensated advanced chronic liver disease and portal hypertension after Baveno VII – an update. Digestive and Liver Disease 55:326–335
- Sharpton SR, Terrault NA, Posselt AM (2019) Outcomes of Sleeve Gastrectomy in Obese Liver Transplant Candidates. Liver Transplantation 25:538–544. https://doi.org/10.1002/lt.25406
- Siegel AB, Lim EA, Wang S, et al (2012) Diabetes, Body Mass Index, and Outcomes in Hepatocellular Carcinoma Patients Undergoing Liver Transplantation. Transplantation 94:539–543. https://doi.org/10.1097/TP.0b013e31825c58ea
- Singal AK, Hasanin M, Kaif M, et al (2016) Nonalcoholic Steatohepatitis is the Most Rapidly Growing Indication for Simultaneous Liver Kidney Transplantation in the United States. Transplantation 100:607–612. https://doi.org/10.1097/TP.0000000000000945
- Soma D, Park Y, Mihaylov P, et al (2022) Liver Transplantation in Recipients With Class III Obesity: Posttransplant Outcomes and Weight Gain. Transplant Direct 8:e1242. https://doi.org/10.1097/TXD.0000000000001242
- Suraweera D, Dutson E, Saab S (2017) Liver Transplantation and Bariatric Surgery: Best Approach. Clin Liver Dis 21:215–230
- Terjimanian MN, Harbaugh CM, Hussain A, et al (2016) Abdominal adiposity, body composition and survival after liver transplantation. Clin Transplant 30:289–294. https://doi.org/10.1111/ctr.12688
- Thuluvath PJ (2007) Morbid Obesity With One or More Other Serious Comorbidities Should Be a Contraindication for Liver Transplantation. Liver Transplantation 13:1627–1629. https://doi.org/10.1002/lt.21211
- Vallin M, Guillaud O, Boillot O, et al (2014) Recurrent or de novo nonalcoholic fatty liver disease after liver transplantation: Natural history based on liver biopsy analysis. Liver Transplantation 20:1064–1071. https://doi.org/10.1002/lt.23936
- Wang G, Huang Y, Yang H, et al (2023) Impacts of bariatric surgery on adverse liver outcomes: a systematic review and meta-analysis. Surgery for Obesity and Related Diseases 19:717–726. https://doi.org/10.1016/j.soard.2022.12.025
- Widmer J, Eden J, Abbassi F, et al (2024) How best to combine liver transplantation and bariatric surgery?—Results from a global, web-based survey. Liver International 44:566–576. https://doi.org/10.1111/liv.15791
- Yemini R, Nesher E, Winkler J, et al (2018) Bariatric surgery in solid organ transplant patients: Long-term follow-up results of outcome, safety, and effect on immunosuppression. American Journal of Transplantation 18:2772–2780. https://doi.org/10.1111/ajt.14739
- Younossi ZM, Koenig AB, Abdelatif D, et al (2016) Global epidemiology of nonalcoholic fatty liver disease-Meta-analytic assessment of prevalence, incidence, and outcomes. Hepatology 64:73–84. https://doi.org/10.1002/hep.28431
- Zamora‐Valdes D, Watt KD, Kellogg TA, et al (2018) Long‐term outcomes of patients undergoing simultaneous liver transplantation and sleeve gastrectomy. Hepatology 68:485–495. https://doi.org/10.1002/hep.29848
- Zhang Y, Ju W, Sun X, et al (2015) Laparoscopic Sleeve Gastrectomy Versus Laparoscopic Roux-En-Y Gastric Bypass for Morbid Obesity and Related Comorbidities: A Meta-Analysis of 21 Studies. Obes Surg 25:19–26. https://doi.org/10.1007/s11695-014-1385-9
- Zhou H, Luo P, Li P, et al (2022) Bariatric Surgery Improves Nonalcoholic Fatty Liver Disease: Systematic Review and Meta-Analysis. Obes Surg 32:1872–1883. https://doi.org/10.1007/s11695-022-06011-1
